# Regulation of RNA Interference Pathways in the Insect Vector *Laodelphax striatellus* by Viral Proteins of Rice Stripe Virus

**DOI:** 10.3390/v13081591

**Published:** 2021-08-11

**Authors:** Yan Xiao, Qiong Li, Wei Wang, Yumei Fu, Feng Cui

**Affiliations:** 1 State Key Laboratory of Integrated Management of Pest Insects and Rodents, Institute of Zoology, Chinese Academy of Sciences, Beijing 100101, China; xiaoyandida@163.com (Y.X.); liqiong@ioz.ac.cn (Q.L.); wangw@ioz.ac.cn (W.W.); 2 College of Life Sciences, Hebei University, Baoding 071002, China; 3 CAS Center for Excellence in Biotic Interactions, University of Chinese Academy of Sciences, Beijing 100049, China; 4 Key Laboratory of Tropical Translational Medicine of Ministry of Education, School of Tropical Medicine and Laboratory Medicine, Hainan Medical University, Haikou 571199, China; fuyumei726@163.com

**Keywords:** siRNA, miRNA, rice stripe virus, planthopper, translin, *Ago2*, viral replication

## Abstract

RNA interference (RNAi), especially the small interfering RNA (siRNA) and microRNA (miRNA) pathways, plays an important role in defending against viruses in plants and insects. However, how insect-transmitted phytoviruses regulate the RNAi-mediated antiviral response in vector insects has barely been uncovered. In this study, we explored the interaction between rice stripe virus (RSV) and the miRNA and siRNA pathways of the small brown planthopper, which is a vector insect. The transcript and protein levels of key genes in the two RNAi pathways did not change during the RSV infection process. When the expression of insect *Ago1*, *Ago2*, or *Translin* was silenced by the injection of double-stranded RNAs targeting these genes, viral replication was promoted with *Ago2* silencing but inhibited with *Translin* silencing. Protein-protein binding assays showed that viral NS2 and RNA-dependent RNA polymerase interacted with insect Ago2 and Translin, respectively. When *NS2* was knocked down, the transcript level of *Ago2* increased and viral replication was inhibited. Therefore, viral NS2 behaved like an siRNA suppressor in vector insects. This protein-binding regulation of insect RNAi systems reflects a complicated and diverse coevolution of viruses with their vector insects.

## 1. Introduction

RNA interference (RNAi) is a sequence-specific gene silencing phenomenon in eukaryotes and is usually induced by small RNA molecules, including small interfering RNAs (siRNAs), microRNAs (miRNAs), and piwi-interacting RNAs (piRNAs) [1,2]. RNAi plays an important role in natural defense against viruses, which act as initiators and as targets of gene silencing, especially in plants and insects [3,4]. During viral infection, the siRNA pathway is activated to target viral genomes for direct degradation or to target host genes for the regulation of viral replication [4,5]. Similarly, miRNAs are produced to regulate the expression of host genes or viral genes through miRNA-viral RNA interactions that affect viral replication [6,7]. Several studies exist regarding the RNAi-mediated anti-viral responses against phytoviruses in plants [3,8,9,10,11,12]. However, the interactions between insect-transmitted phytoviruses and the RNAi pathways of vector insects remain elusive. Understanding these interactions would facilitate the control of insects that transmit these plant viruses through regulating specific RNAi pathways or the expression of certain small RNA molecules to interrupt viral infection.

In insects, the main factors in the miRNA pathway include Drosha (RNaseIII), Pasha (double-stranded RNA binding protein), Dicer1, and Argonaute1 (Ago1); Dicer2 and Ago2 are the major players in the siRNA pathway [13,14]. Translin also plays an important role in RNAi. Through interaction with a strong binding factor, Trax, Translin and Trax form the C3PO complex, which inhibits the synthesis of miRNAs by degrading pre-miRNAs [15,16]. Interestingly, the CP3O complex has also been found to promote the siRNA pathway by assisting Ago2 in degrading the passenger strands of siRNA duplexes [17]. In our recent work, we predicted the interactions between viral proteins of rice stripe virus and the Ago2 and Translin of insect vectors [18].

Rice stripe virus (RSV) is a nonenveloped, negative-strand RNA virus that causes serious rice stripe disease in Asian countries [19]. The genome of RSV consists of four RNA segments, encoding one nucleocapsid protein (NP), one RNA-dependent RNA polymerase (RdRp), and five nonstructural proteins (NS2, NSvc2, NS3, SP, and NSvc4) [20,21,22]. RSV is efficiently transmitted by the small brown planthopper *Laodelphax striatellus* in a persistent-propagative manner [23]. We have previously revealed the complicate interactions between RSV and the innate immune systems of the vector insect such as the c-Jun N-terminal kinase pathway [24], prophenoloxidase activation pathway [25], and apoptosis [26]. How RSV interacts with the miRNA and siRNA pathways of the vector insect to maintain a tolerable replication level has not been fully explored.

In this study, we evaluated the response and roles of the miRNA and siRNA pathways to RSV infection at the transcriptomic and protein levels in small brown planthoppers. Our work demonstrates the absence of significant gene expression variation of key RNAi genes in RSV-infected planthoppers. However, interfering with *Ago2* promoted RSV replication, while interfering with *Translin* was detrimental to RSV replication. Protein-protein binding assays showed that NS2 and RdRp of RSV interacted with Ago2 and Translin, respectively, indicating that the virus regulated these two RNAi pathways in a way of protein-protein interactions.

## 2. Materials and Methods

### 2.1. Planthoppers and Rice Viruses

The viruliferous and nonviruliferous small brown planthopper strains were reared separately in the laboratory on seedlings of rice, *Oryza sativa* Huangjinqing, at 25 °C and 16 h of light daily, as described previously [27]. The viruliferous strain harbored the Jiangsu RSV isolate, and the frequency of RSV positivity was maintained at no less than 90% through purification selection performed every three months via dot-ELISA with a monoclonal anti-NP antibody [27].

### 2.2. RNA Isolation and cDNA Synthesis

Total RNA from a pool of five planthoppers sampled in different experiments for quantitative real-time PCR was isolated using TRIzol Reagent (Invitrogen, Carlsbad, CA, USA) according to the manufacturer’s instructions. The quality and concentration of RNA were measured using a NanoDrop 2000 spectrophotometer (Thermo Scientific, Waltham, MA, USA). Two micrograms of total RNA were applied in first-strand cDNA synthesis using MLV reverse transcriptase (Promega, Madison, WI, USA) and random primers (Promega) according to the manufacturer’s instructions. The cDNA samples were used as templates in quantitative real-time PCRs.

### 2.3. Injection of RSV Crude Preparations

Approximately 60 viruliferous adults of *L. striatellus* with a 1:1 sex ratio at the age of 4 to 6 d after emergence were homogenized using a disposable polypropylene pestle in 100 μL of 10 mM PBS (pH 7.4) in a 1.5 mL tube. Centrifugation at 12,000× *g* for 15 min at 4 °C was repeated four times, and the supernatant from the last round of centrifugation was retained as the RSV crude preparation. A 23 nL aliquot of the RSV crude preparation was injected into nonviruliferous third-instar nymphs through a glass needle using a Nanoliter 2000 microinjector (World Precision Instruments, Sarasota, FL, USA). Insects were collected at 2, 4, 6, 8, and 10 d after injection. Gene expression was determined by qPCR and western blot. Five planthoppers were contained in one replicate, eight biological replicates were prepared for qPCR, and three replicates were prepared for western blot.

### 2.4. Synthesis of dsRNAs

PCR primers with the T7 promoter sequences *AGO1*-dsRNA-F/*AGO1*-dsRNA-R, *AGO2*-dsRNA-F/*AGO2*-dsRNA-R, *translin*-dsRNA-F/*translin*-dsRNA-R, and *NS2*-dsRNA-F/*NS2*-dsRNA-R were used to generate 337 bp of *AGO1* dsRNA, 161 bp of *AGO2* dsRNA, 279 bp of *NS2* dsRNA, and 498 bp of *Translin* dsRNA. A segment of 420 bp of GFP was amplified using primers GFP-dsRNA-F/GFP-dsRNA-R dsRNA as a negative control (Table S1). All dsRNAs were synthesized using the T7 RiboMAX Express RNAi System (Promega) following the manufacturer’s protocol. Briefly, 1 μg of forward or reverse DNA template with a T7 linker, 10 μL of RiboMAX Express T7 2× Buffer, and 2 μL of enzyme mix were mixed in a final volume of 20 μL and incubated at 37 °C for 3 h to produce the complementary single-stranded RNA. Equal volumes of complementary single-stranded RNA were put together to form dsRNA, which was extracted with phenol chloroform and precipitated with a solution containing 0.1 volume of 3 M sodium acetate (pH 5.2) and 2.5 volume of 95% ethanol. The dsRNA precipitates were washed with 75% ethanol and dissolved in RNase-free water.

### 2.5. Injection of dsRNAs

A volume of 23 nL of dsRNA at 6 μg/μL or a mixture of 12 μg/μL dsRNA and crude RSV preparation at a ratio of 1:1 was injected into viruliferous or nonviruliferous third-instar nymphs through a glass needle using a Nanoliter 2000 microinjector (World Precision Instruments). The planthoppers were raised on healthy rice seedlings for 6 d after injection. RNA was isolated and gene expression was determined by qPCR and western blot. Five planthoppers were contained in one replicate, eight biological replicates were prepared for qPCR, and four replicates were prepared for western blot.

### 2.6. Quantitative Real-Time PCR

Eighty-five base pairs of *Drosha*, 166 bp of *Pasha*, 82 bp of *Dicer1*, 89 bp of *Dicer2*, 201 bp of *Ago1*, 175 bp of *Ago2*, 149 bp of *Translin*, 99 bp of *Trax*, 165 bp of *NS2*, 60 bp of *NP,* and 92 bp of RSV RNA3 were amplified by qPCR. Sixty-four base pairs of *elongation* *factor 2* (*EF2*) of *L. striatellus* were amplified as an internal reference. The primers are listed in Table S1. The reaction mix of qPCR was 10 μL, which included 1 μL of cDNA template, 5 μL of SYBR Green I Master Mix (Roche, Basel, Switzerland), and 0.25 μL of primers with a concentration of 10 μM. The qPCR was performed on a LightCycler 480^®^ II (Roche) with the thermal cycling conditions of 95 °C for 2 min, 40 cycles of 95 °C for 20 s, 58 °C for 20 s, and 68 °C for 20 s, followed by one cycle of 95 °C for 30 s, 58 °C for 30 s, and 95 °C for 10 s to construct the melting curve. The relative RNA or transcript level of each gene was calculated by 2^–ΔCt^ method, where ΔCt is the Ct value (threshold cycle) of the target gene minus that of *EF2*, and it was reported as the mean ± SE. Differences were statistically evaluated using Student’s *t*-test to compare two means and one-way ANOVA followed by Tukey’s test for multiple comparisons in SPSS 17.0. 

### 2.7. Western Blot Assay

Total proteins from planthoppers were extracted using TRIzol Reagent (Invitrogen) along with RNA extraction. The protein levels of NP, Ago1, Ago2, Translin, and Tubulin were measured by western blot using homemade monoclonal antibodies for NP, Ago1, Ago2 [28], and polyclonal antibody for Translin and commercial monoclonal anti-β-tubulin antibody (CWBIO, Beijing, China) as the primary antibodies. Goat anti-mouse IgG or goat anti-rabbit IgG (CWBIO) was used at 1:10,000 as a secondary antibody. Immune signals were displayed by chemiluminescence (ECL kit, Thermo Scientific) and visualized using Image Station 4000 MM ProCFL (Carestream, Rochester, NY, USA). The relative grayscales of NP, Ago1, Ago2, and Translin to that of tubulin were quantified by ImageJ software and compared statistically between different groups by Student’s *t*-test in SPSS 17.0 software.

### 2.8. Recombinant Protein Expression in Escherichia coli and Antibody Preparation

The full-length open reading frames of *Translin* and viral *NS2* and *RdRp* fragment 2 from 482 to 1051 aa were cloned from the cDNA library of viruliferous planthoppers. *Translin* and *NS2* were inserted into the pET28a vector between the restriction sites *Nco*I and *Xho*I to generate recombinant plasmids with His-tags. *RdRp2* was inserted between the restriction sites *EcoR*I and *Xho*I of the pET28a vector to generate the recombinant plasmid with His-tags. The primers used for this purpose are listed in Table S1.

The recombinant plasmids were transformed into *E. coli* strain BL21 (DE3) for protein expression. The cells were induced with 0.5 mM isopropyl β-D-thiogalactoside (IPTG) at 28 °C for 12 h before being pelleted by centrifugation and sonicated for 30 min in ice water. The supernatant of the cells expressing NS2-His or RdRp2-His was retained for Co-IP assays, and that of the cells expressing Translin-His was retained for protein purification using Ni Sepharose (GE Healthcare, Buckinghamshire, UK) following the manufacturer’s instructions. The purified Translin-His served as the antigen to produce an anti-Translin rabbit polyclonal antibody at the Beijing Protein Institute Co., Ltd. (Beijing, China).

### 2.9. Coimmunoprecipitation Assay

Five micrograms of anti-His monoclonal antibody was first incubated with 50 μL of Dynabeads^®^ Protein G (Novex^®^, Thermo Fisher Scientific, Waltham, MA, USA) for 30 min, after which 300 μL of NS2-His or RdRp2-His was added and incubated for 1 h at 4 °C. After washing three times with washing buffer (Novex^®^), 400 μL of total protein from viruliferous planthoppers in 10 mM phosphate buffer (pH 7.2) was added and incubated with the bead-antibody-protein complex for 1 h at 4 °C. Approximately 10% of the total protein was reserved as input. Mouse IgG (Merck Millipore, Billerica, MA, USA) was used as a negative control. After washing three times with washing buffer, the antibody-protein complex was disassociated from the beads with elution buffer (Novex^®^) for western blot analysis using anti-Ago2 and anti-His monoclonal antibodies as well as the anti-Translin polyclonal antibody.

## 3. Results

### 3.1. Response of miRNA and siRNA Pathways to RSV Infection in Planthoppers

**Figure 1:**
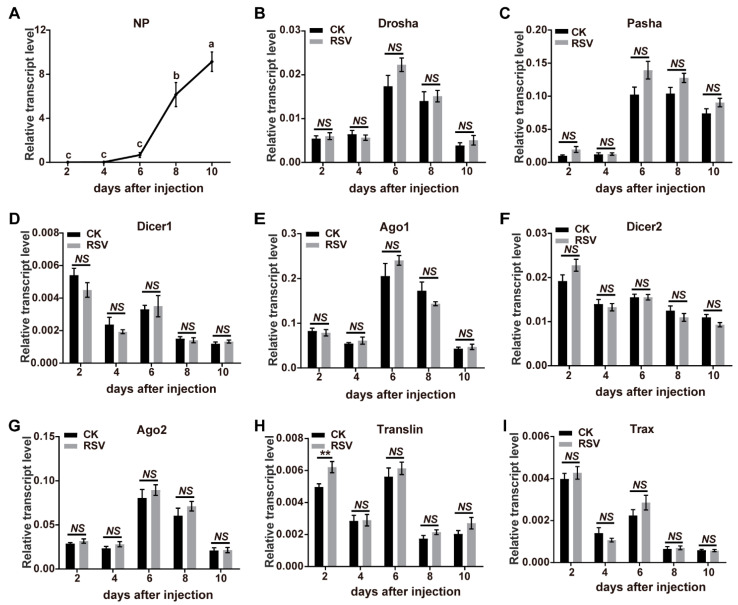
Response of miRNA and siRNA pathways to RSV infection in planthoppers. (**A**) The relative RNA levels of RSV *NP* in the planthoppers after injection of RSV crude preparations from viruliferous planthoppers measured with quantitative real-time PCR (qPCR). Significant differences are indicated by different lowercase letters. (**B**–**I**) The relative transcript levels of *Drosha*, *Pasha*, *Dicer1*, *Ago1*, *Dicer2*, *Ago2*, *Translin,* and *Trax* in the miRNA and siRNA pathways during the infection process of RSV in the planthoppers were measured with qPCR. The crude preparations from nonviruliferous planthoppers were injected into the control groups (CK). The RNA or transcript levels of these genes were normalized to that of *EF2*. The values represent the means ± SEs. NS, no significant difference. **, *p* < 0.01.

**Figure 2:**
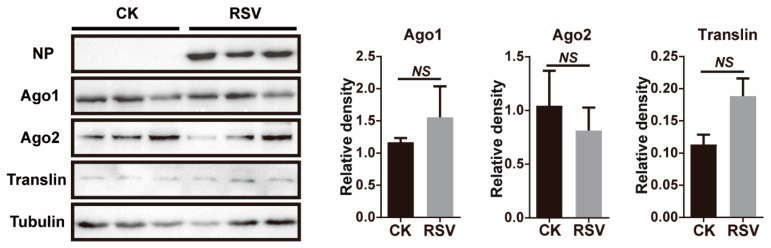
Western blot assay showing the protein levels of NP, Ago1, Ago2, and Translin in the planthoppers at 6 d after injection of RSV crude preparations. The crude preparations from nonviruliferous planthoppers were injected into the control group (CK). Three biological replicates were shown for each group. The monoclonal antibodies for NP, Ago1 and Ago2, and polyclonal antibody for Translin were used. The protein level of planthopper tubulin was revealed using the monoclonal anti-β-tubulin antibody as an internal reference. The relative grayscales of Ago1, Ago2, and Translin to that of tubulin were quantified and compared between the RSV-infected and CK groups. The values represent the means ± SEs. NS, no significant difference.

To determine if RSV infection led to transcriptomic changes in 8 key genes of the miRNA and siRNA pathways, infected planthoppers were used to measure transcript levels via quantitative real-time PCR (qPCR). *Ago1*, *Ago2*, *Dicer1*, *Dicer2*, *Drosha*, *Pasha*, *Translin*, and *Trax* were identified from the gene set of the small brown planthopper [29]. When RSV crude preparations from viruliferous planthoppers were injected into nonviruliferous planthoppers, viral replication was observed from 6 d after injection (DAI) as shown by an increase in *NP* RNA levels (Figure 1A, *F*_(4, 35)_ = 43.5, *p* < 0.0001). The relative transcript levels of the eight genes in the RSV-infected planthoppers were measured and compared to those of nonviruliferous planthoppers injected with crude preparations from nonviruliferous insects at 2, 4, 6, 8, and 10 DAI. qPCR results demonstrate a lack of significant changes compared to the controls in seven of the eight genes measured at most of time points of viral infection (Figure 1B–I, *p* > 0.05, df from 9 to 14). However, a significant increase of *Translin* was detected at 2 DAI (Figure 1H, *p* < 0.01, df = 12). Considering that Ago1 and Ago2 are key components of the RNA-induced silencing complex (RISC) and Translin functions in both of miRNA and siRNA pathways, we further evaluated the protein levels of the three genes at 6 DAI with RSV infection by western blot using homemade monoclonal antibodies for Ago1 and Ago2 [28] and a polyclonal antibody for Translin. The results showed that no significant variation was observed for Ago1 and Ago2 (Figure 2, *p* > 0.05, df = 4) and a marginally significant increase for Translin (Figure 2, *p* = 0.075, df = 4). Therefore, the miRNA and siRNA pathways of the vector insects almost did not obviously respond to RSV infection in terms of alterations in gene expression levels.

### 3.2. Roles of miRNA and siRNA Pathways in the Control of RSV Replication

**Figure 3:**
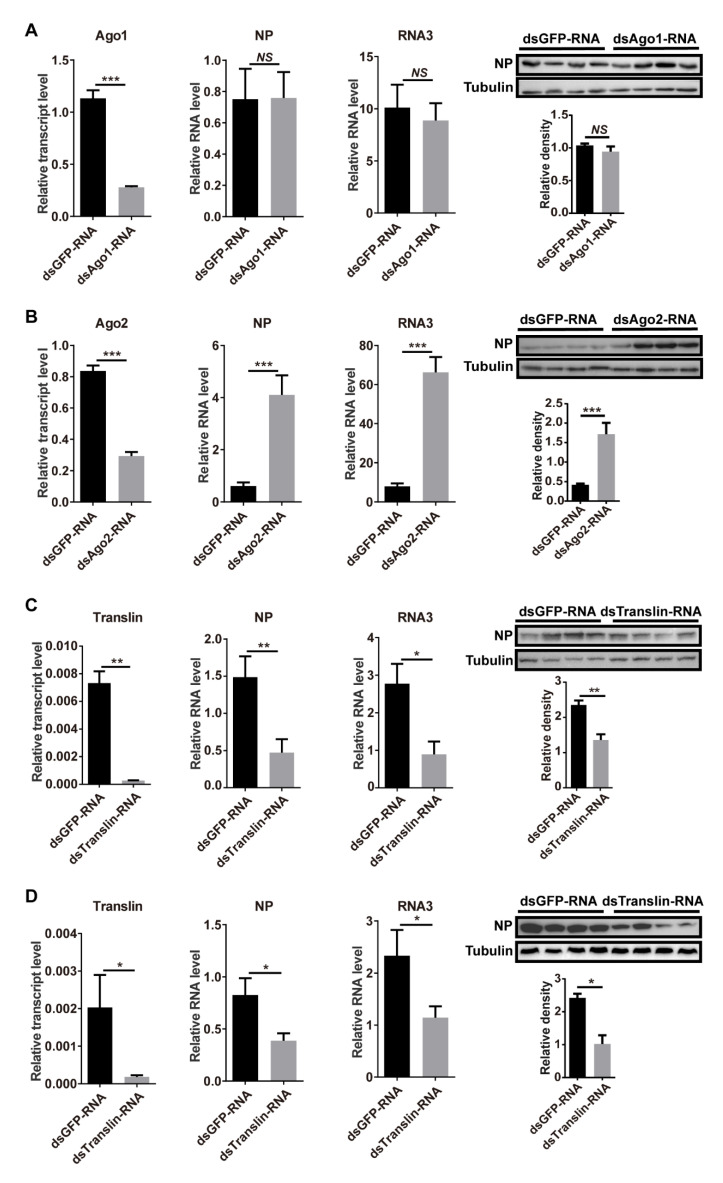
Roles of miRNA and siRNA pathways in the control of RSV replication. (**A**–**C**) Effect of planthopper *Ago1*, *Ago2*, and *Translin* transcript levels, and viral *NP* and RNA3 levels at 6 d after injection of double-stranded RNAs (dsRNAs) with RSV crude preparations. The control group was injected with ds*GFP*-RNA and RSV crude preparations. (**D**) Effect of planthopper *Translin* transcript level and viral *NP* and RNA3 levels in the stable viruliferous strain at 6 d after injection of ds*Translin*-RNA. The control group was injected with ds*GFP*-RNA. The relative RNA levels of *NP* and RNA3 to that of *EF2* and relative transcript levels of the three planthopper genes to that of *EF2* were quantified with quantitative real-time PCR. The protein levels of NP and tubulin were measured using monoclonal anti-NP and anti-β-tubulin antibodies by western blot. Four biological replicates were shown for each group. The relative grayscale of NP to that of tubulin was quantified and compared between the gene interference groups and ds*GFP*-RNA injection groups. The values represent the means ± SEs. *, *p* < 0.05. **, *p* < 0.01. ***, *p* < 0.001. NS, no significant difference.

To explore the functions of miRNA and siRNA pathways in RSV replication, we silenced the expression of *Ago1*, *Ago2*, or *Translin* by injection of double-stranded RNAs (dsRNAs), targeting these genes with RSV crude preparations. Compared to the control group that was injected with ds*GFP*-RNA and RSV crude preparations, the expression of *Ago1*, *Ago2*, or *Translin* was successfully knocked down with 75% (*p* < 0.001, df = 14), 65% (*p* < 0.001, df = 14), or 96% (*p* < 0.01, df = 14) decreases in transcript levels (Figure 3A–C). Knockdown of *Ago1* did not affect the RNA levels of *NP* (*p* > 0.05, df = 14) and RNA3 (*p* > 0.05, df = 14) or the NP protein level (*p* > 0.05, df = 6) (Figure 3A) at 6 DAI. Knockdown of *Ago2* promoted the RSV amount in terms of the RNA levels of *NP* (*p* < 0.001, df = 14) and RNA3 (*p* < 0.001, df = 14) as well as the NP protein level (*p* < 0.001, df = 6) (Figure 3B). These results indicated that inhibition of the siRNA pathway facilitated RSV replication, while inhibition of the miRNA pathway had no obvious influence on RSV replication. When *Translin* was knocked down in the RSV-injected planthoppers (*p* < 0.01, df = 14), the RNA levels of *NP* (*p* < 0.01, df = 14) and RNA3 (*p* < 0.05, df = 14) and the NP protein level (*p* < 0.01, df = 6) significantly decreased at 6 DAI (Figure 3C). Similarly, when *Translin* was knocked down in individuals from the stable viruliferous strain (*p* < 0.05, df = 13), the RNA levels of *NP* (*p* < 0.05, df = 13) and RNA3 (*p* < 0.05, df = 13) and the NP protein level (*p* < 0.05, df = 6) significantly decreased at 6 DAI (Figure 3D). This showed that Translin played a positive role in RSV replication in the planthoppers.

### 3.3. Regulation of RNAi Pathways through Insect Protein-Viral Protein Interactions

**Figure 4:**
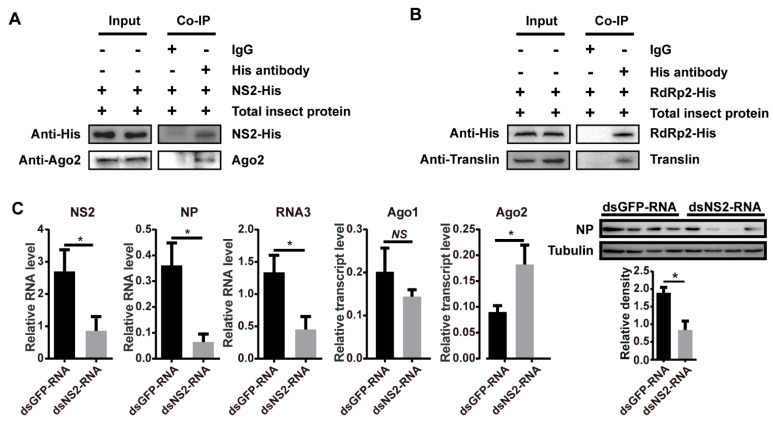
Regulation of RNAi pathways through insect protein-viral protein interactions. (**A**) Coimmunoprecipitation (Co-IP) assay demonstrating that the recombinant expressed NS2-His pulled down Ago2 from the crude extracts of viruliferous planthoppers. (**B**) Co-IP assay demonstrating that the recombinant expressed RdRp2-His pulled down Translin from the crude extracts of viruliferous planthoppers. Monoclonal antibodies against Ago2 and His and polyclonal antibodies against Translin were applied. IgG was used as a negative control. (**C**) Effect of planthopper *Ago1* and *Ago2* transcript levels and viral *NS2*, *NP,* and RNA3 levels in the planthoppers at 6 d after injection of double-stranded RNAs (dsRNAs) of *NS2* with RSV crude preparations. The control group was injected with ds*GFP*-RNA and RSV crude preparations. The relative transcript or RNA levels to that of *EF2* were quantified with quantitative real-time PCR. The protein levels of NP and tubulin were measured using monoclonal anti-NP and anti-β-tubulin antibodies by western blot. Four biological replicates were shown for each group. The relative grayscale of NP to that of tubulin was quantified and compared between the gene interference group and ds*GFP*-RNA injection groups. The values represent the means ± SEs. *, *p* < 0.05. NS, no significant difference.

In our recent work, we predicted protein-protein interactions between five rice viruses (RSV, rice black-streaked dwarf virus, rice grassy stunt virus, rice ragged stunt virus, and southern rice black-streaked dwarf virus) and their respective vector insects (*L. striatellus*, *Nilaparvata lugens*, and *Sogatella furcifera*) on a genome-wide scale using the DeNovo method [18]. Ago2 and Translin were predicted to be able to bind NS2 and RdRp of RSV, respectively, and the in vitro pulldown assay showed that translin bound fragment 2 from amino acid (aa) 482 to 1051 of RdRp [18]. To further verify these protein-protein interactions, NS2 and fragment 2 of RdRp (RdRp2) were recombinantly expressed with His-tags. The coimmunoprecipitation (co-IP) assays demonstrated that NS2-His pulled down Ago2 (Figure 4A) and RdRp2-His pulled down Translin from viruliferous planthoppers (Figure 4B), confirming the interactions between NS2 and Ago2 and between RdRp and Translin.

NS2 has been identified as an RNAi suppressor in viral host plants [30]. To demonstrate whether NS2 also functions as an RNAi suppressor in the planthoppers, we injected ds*NS2*-RNA and RSV crude preparations into nonviruliferous planthoppers. Compared to the control group, which was injected with ds*GFP*-RNA and RSV crude preparations, the RNA level of *NS2* was knocked down (*p* < 0.05, df = 16), and the replication of RSV was inhibited, as shown by the decreased RNA levels of *NP* (*p* < 0.05, df = 16) and RNA3 (*p* < 0.05, df = 16) as well as the NP protein level (*p* < 0.05, df = 6) at 6 DAI (Figure 4C). Furthermore, the transcript level of *Ago2* increased (*p* < 0.05, df = 16) while no change was observed for *Ago1* (*p* > 0.05, df = 16) with the knockdown of NS2 (Figure 4C). The restricted viral replication due to a lack of NS2 was consistent with the antiviral effect of the Ago2-mediated siRNA pathway, reflecting a possible of role of NS2 as an RNAi suppressor in the planthoppers. The binding of NS2 with Ago2 may disturb the role of Ago2 in the siRNA pathway and be helpful for viral replication.

## 4. Discussion

As a persistent and propagative phytovirus, RSV has a limited level of replication in vector insects compared to host plants. This limited viral load in vector insects is the result of the balance between viruses and insect immune systems. Since RNAi is the main insect antiviral immune system, the interaction between RSV and the RNAi system could play a vital role in maintaining a tolerable viral titer in vector insects. Here, we found that RSV did not significantly affect the expression levels of genes involved in the miRNA and siRNA pathways in the vector insects. Instead, the interactions between viral proteins and essential proteins of RNAi pathways affected the two RNAi pathways. On the one hand, the siRNA pathway was probably inhibited by viral NS2 protein to be beneficial for viral replication. On the other hand, binding to viral RdRp by Translin was disadvantageous to viral replication. This protein-binding regulation of insect RNAi immune systems to produce a seesaw-like effect may be a specific strategy for RSV to maintain a tolerable replication level in vector insects.

The regulation of insect RNAi pathways by RSV is quite unique. In our previous transcriptomic work, we found that *Dicer1*, *Dicer2*, *Ago1*, *Ago2*, and *Ago3* did not change in terms of transcript levels upon RSV infection in salivary glands or the gut of small brown planthoppers [27]. Similar results were reported in the whole bodies of viruliferous small brown planthoppers compared to naïve samples based on comparative EST analysis [31]. However, activation of the RNAi pathways is frequently observed for arthropod-borne phytoviruses or human viruses in vector insects. For example, the expression of *Dicer2* and *Ago2* was significantly upregulated in the leafhopper *Recilia dorsalis* with rice gall dwarf virus infection [32]. When the titer of the southern rice black streaked dwarf virus increased in *S. furcifera*, the expression of 15 RNAi-related genes, including *Dicer1*, *Dicer2*, *Ago1,* and *Ago3*, was enhanced [33]. Infection with DENV in *Aedes aegypti* induced the overexpression of *Dicer2* and *Ago2* [34]. In contrast to these insect-transmitted viruses, we found that RSV applied a unique strategy of protein-protein interactions to manipulate the RNAi pathways. The nonstructural protein NS2 and the RdRp of RSV directly bind Ago2 and Translin, respectively, to affect the antiviral defense of RNAi pathways.

Suppression of the miRNA pathway seems not as effective for RSV replication as suppression of the siRNA pathway is. We found that decreasing *Ago2* expression, not *Ago1* expression, leads to a significant promotion of RSV replication. Similarly, interfering with *Ago1* expression in the vector *Anopheles gambiae* did not lead to a significant variation of the titer of the O’nyong-nyong virus [35]. Silencing *Dicer1* and *Ago1* genes in the leafhopper *R. dorsalis* did not affect the titer of the rice gall dwarf virus [32]. In the *Dicer1* mutant strain of *Drosophila melanogaster*, there was no significant change in susceptibility to Drosophila X virus infection [4]. On the other hand, silencing *Dicer1* and *Ago1* in Sf9 insect cells improved replication levels of Autographa californica multiple nucleopolyhedrovirus [36]. The uncertain effects of the miRNA pathway on virus infection in insects could be related to the indirect regulation of miRNAs to viruses through targeting insect genes [37]. It is generally deemed that siRNAs are predominantly responsible for antiviral activity in insects through targeting viral genomes for direct degradation [4].

We found that RSV NS2 behaved like an siRNA suppressor in vector insects. NS2 has been identified as an RNAi suppressor in viral host plants. Plant RNA-dependent RNA polymerases (RDRs) have a pivotal role in mediating resistance to many plant viruses. Suppressor of gene silencing 3 (SGS3) interacts with RDR6 to trigger endogenous silencing in *Nicotiana benthamiana* [38]. RSV NS2 inhibits RNA silencing by binding with rice OsSGS3 to deactivate the RDR-SGS3 siRNA pathway [30]. In planthoppers, NS2 directly binds Ago2 and reduces the expression of Ago2 to downregulate the antiviral defense of the siRNA pathway. The RSV NS3 protein also works as an RNAi suppressor in rice. NS3 regulates the rice miRNA pathway by interacting with double-stranded RNA-binding protein 1 (OsDRB1), which is necessary for the formation of mature miRNAs [39]. However, based on our bioinformatic prediction of protein-protein interactions between rice viruses and their vector insects [18], it is unlikely that NS3 binds Ago or Dicer proteins of the planthoppers. As RNAi suppressors, NS2 and NS3 function differently both in vector insects and in host plants.

Translin plays a positive role in RSV replication in insect vectors. Translin is a single-stranded DNA/RNA binding protein [40]. Although Translin works in both the miRNA and siRNA pathways with opposite effects as a member of the C3PO complex [15,16,17], it may act as a miRNA suppressor during RSV infection. Otherwise, interference of *Translin* expression using dsRNAs should not produce an antiviral reaction. However, it is not clear whether the interaction between RSV RdRp and Translin activates or inhibits the miRNA pathway. Identification of the miRNAs that are overexpressed and the genes targeted by these miRNAs requires further exploration with interference of *Translin* expression.

In conclusion, unlike the frequent activation of the RNAi pathways in vector insects by phytoviruses or human viruses through provoking RNAi gene expression changes, RSV targets the RNAi systems of vector insects via interactions of viral proteins and RNAi proteins. This unusual regulation strategy for insect RNAi systems reflects a complicated and diverse coevolution phenomenon of viruses with their vector insects.

## Data Availability

The gene sequences used in this study are openly available with the following registration numbers for *Ago1* (evm.model.Contig18.206.10), *Ago2* (evm.model.Contig47.47), *Dicer1* (evm.model.Contig227.50), *Dicer2* (evm.model.Contig30.130), *Drosha* (evm.model.Contig18.256), *Pasha* (evm.model.Contig18.80), *Translin* (evm.model.Contig223.14), *Trax* (evm.model.Contig256.29), and *EF2* (evm.model.Contig0.299) in the geneset of the small brown planthopper (GigaScience Database 2 November 2017. http://dx.doi.org/10.5524/100361); RSV *NP* (DQ299151), RNA3 (MF287953.1), *NS2* (EF493228), and *RdRp* (JQ927433) in the GenBank.

## Supplementary Materials

The following are available online at https://www.mdpi.com/article/10.3390/v13081591/s1, Table S1: Primers used in this study.
