# Supplementary material for: Regulation of RNA Interference Pathways in the Insect Vector Laodelphax striatellus by Viral Proteins of Rice Stripe Virus
Source: Viruses. 2021 Aug 11;13(8):1591. doi: 10.3390/v13081591 (PMC8402809; doi:10.3390/v13081591)
Supplement: Supplementary file 1 [file viruses-13-01591-s001.zip › viruses-1301071-supplementary.pdf]

Table S1. Primers used in this study

Primer name	Primer sequence (5’ to 3’)
GFP-RNAi-F	CACAAGTTCAGCGTGTCCG
GFP-RNAi-R	GTTCACCTTGATGCCGTTC
GFP-RNAi-T7F	TAATACGACTCACTATAGGCACAAGTTCAGCGTGTCCG
GFP-RNAi-T7R	TAATACGACTCACTATAGGGTTCACCTTGATGCCGTTC
NS2-F	ATGGCATTACTCCTTTTCAATGA
NS2-R	TCACATTAGAATAGGACACTC
NS2-RNAi-F	TGAAGCTGATCTTGTTGCCCA
NS2-RNAi-R	ACTCATGTGCTCCGACCAAG
NS2-RNAi-T7F	TAATACGACTCACTATAGGTGAAGCTGATCTTGTTGCCCA
NS2-RNAi-T7R	TAATACGACTCACTATAGGACTCATGTGCTCCGACCAAG
NS2-qPCR-F	GCTCTCGCCATAGCAACTGA
NS2-qPCR-R	AGCAAACTGAAACCCGTTGAC
AGO1-RNAi-F	TGCCAATTATCGGCCAACCT
AGO1-RNAi-R	CCCAGGAATATCACCGGCTC
AGO1-RNAi-T7F	TAATACGACTCACTATAGGTGCCAATTATCGGCCAACCT
AGO1-RNAi-T7R	TAATACGACTCACTATAGGCCCAGGAATATCACCGGCTC
AGO1-qPCR-F	GGCCTCACCATCTCCAACAA
AGO1-qPCR-R	CTCGACGCCAGTGAAGAACT
AGO2-RNAi-F	AACCGCAAACTGACTCCGAT
AGO2-RNAi-R	GCGAATCTGTCCGATACCGT
AGO2-RNAi-T7F	TAATACGACTCACTATAGGAACCGCAAACTGACTCCGAT
AGO2-RNAi-T7R	TAATACGACTCACTATAGGGCGAATCTGTCCGATACCGT
AGO2-qPCR-F	TAAACCGGACAAGCCTCGAC
AGO2-qPCR-R	CTGCCGTTCGCTGTTGAAAA
Translin-F	ATGGATTTAGATTTTAAATCG
Translin-R	CTAACTCTTATTCTCCGTTG
Translin-RNAi-F	TGCTCGAAGGCTCGTGAA
Translin-RNAi-R	ATTCTCCGTTGAAGTCAAGTGT
Translin-RNAi-T7F	TAATACGACTCACTATAGGTGCTCGAAGGCTCGTGAA
Translin-RNAi-T7R	TAATACGACTCACTATAGGATTCTCCGTTGAAGTCAAGTGT
Translin-qPCR-F	TGAAGAATGATGACGAGCGTTT
Translin-qPCR-R	TCACGAGCCTTCGAGCAATA
Drosha- qPCR -F	CGGTAGCAGTAGTAGCAATTCA
Drosha- qPCR -R	TTCTCGCCAGCATAGATGTTAT
Pasha-qPCR-F	CGTAGTGGAATGCCTCTC
Pasha-qPCR-R	GTGCCTTCTTCTGCTCTT
Trax-qPCR-F	CGGACGATATTGTTGAGGACTC
Trax-qPCR-R	TTGAGGCATCAGTGGAGACC
RdRp2-EcoRI-F	TCGCGGATCCGAATTCGAGGCTGTTGATGTAAAGAC
RdRp2-XhoI-R	GGTGGTGGTGCTCGAGTCATGATTCTGGACTGCCTATC

Translin-NcoI-F	AGGAGATATACCATGGGCATGGATTTAGATTTT
Translin-XhoI-R	GGTGGTGGTGCTCGAGACTCTTATTCTCC
NS2-NcoI-F	AGGAGATATACCATGGGCATGGCATTACTCCT
NS2-XhoI-R	GGTGGTGGTGCTCGAGCATTAGAATAGGACACTC
